# Supplementary material for: TRIM16 Overexpression in HEK293T Cells Results in Cell Line-Specific Antiviral Activity
Source: Pathogens. 2023 Jun 20;12(6):852. doi: 10.3390/pathogens12060852 (PMC10301484; doi:10.3390/pathogens12060852)
Supplement: Supplementary file 1 [file pathogens-12-00852-s001.zip › pathogens-2220182-supplementary.pdf]

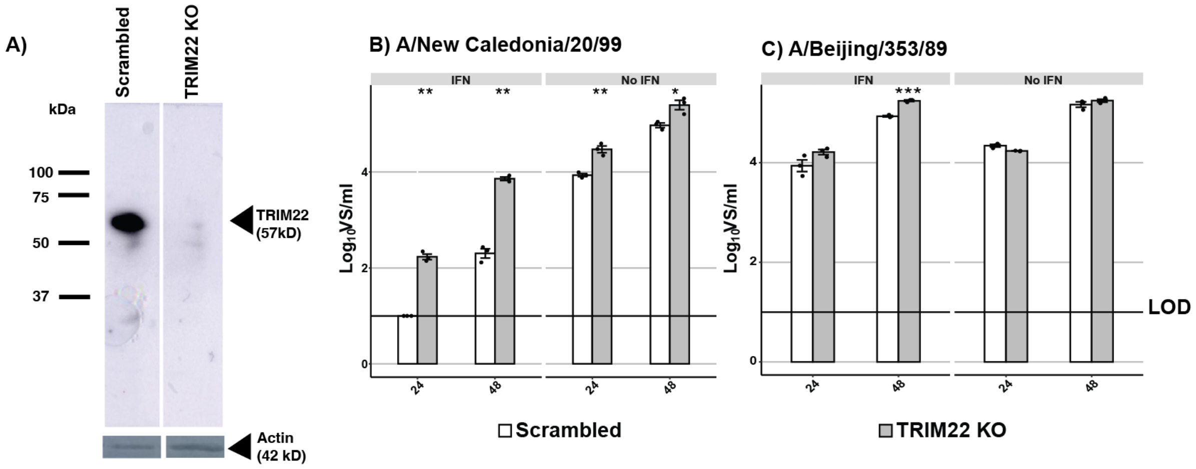

Figure S1. Confirming the antiviral activity of endogenous TRIM22 against influenza A virus in A549 cells using CRISPR/Cas9 editing. (**A**) A549 TRIM22 knockout (TRIM22 KO) and control (Scrambled) cells was generated using CRISPR/Cas9 editing. TRIM22 protein expression in bulk A549 cell populations treated with 1000u/mL IFN-α for 24 hr and was then assessed by western blot using a TRIM22-specific pAb. Actin is included as a loading control. (**B–C**) Bulk TRIM22 KO or Scrambled A549 cell populations were either mock-treated or pre-treated with 1000 units/mL IFN-α for 24 hr, washed and then infected with (**B**) IAV strain A/New Caledonia/20/99 (H1N1, MOI 0.01), or (**C**) A/Beijing/353/89 (H3N2, MOI 0.1). At 24 and 48 hpi supernatants were collected and titres of infectious virus determined by VS assay on MDCK cells. Representative data from one of two independent experiments are shown. Statistical analyses were performed using an unpaired two-sample Student's t-test. The p-values are indicated as follows: * <0.5, **<0.1,***<0.01, **** <0.001. LOD- limit of detection.
